# Changes in the concentration of phosphatidylcholine in lipid bilayers determines the aggregation rate of transthyretin

**DOI:** 10.1016/j.bpc.2026.107574

**Published:** 2026-01-08

**Authors:** Abid Ali, Mikhail Matveyenka, Dmitry Kurouski

**Affiliations:** Department of Biochemistry and Biophysics, Texas A&M University, College Station, TX 77843, United States

**Keywords:** Transthyretin, Phospholipids, ROS, Fibrils

## Abstract

Transthyretin (TTR) is a tetrameric transporter of retinol and thyroxine that aggregates in the central and peripheral nervous system upon a severe pathology known as transthyretin amyloidosis. Although small molecular weight drugs can stabilize TTR preventing its aggregation, molecular mechanisms of transthyretin amyloidosis remain poorly understood. Accumulating evidence indicates that lipids can alter TTR stability by facilitating protein aggregation into toxic oligomers and fibrils. Consequently, pathological changes in the lipid composition of plasma membranes can be responsible for the onset and progression of transthyretin amyloidosis. In this study, we investigated the role of concentration-dependent changes in phosphatidylcholine (PC), one of the most abundant phospholipids in the plasma membrane, on the rate of TTR aggregation. For this, TTR was exposed to large unilamellar vesicles (LUVs) composed of 30%, 35%, and 40% PC. We found that a decrease in the concentration of PC from 40% to 35% drastically accelerated TTR aggregation. We also observed an increase in the cytotoxicity of TTR aggregates formed in the presence of 35% PC compared to TTR fibrils grown in the presence of LUVs with 40% PC. These results indicate that changes in the concentration of PC in the plasma membrane could trigger amyloid formation that leads to transthyretin amyloidosis.

## 1. Introduction

Transthyretin amyloidosis is caused by the progressive accumulation of transthyretin (TTR), retinol and thyroxine transporter, in various parts of the body, including brain, heart, central and peripheral nervous system [1–9]. During this pathological process, tetrameric protein splits into monomers that aggregate forming highly toxic oligomers and fibrils [10–14]. Our group showed that the toxicity of TTR aggregates could be altered by lipid vesicles present at the stage of protein aggregation [15–18]. Specifically, phosphatidylcholine (PC) significantly reduced the cytotoxicity of amyloid fibrils formed by TTR, as well as decelerated the rate of TTR aggregation. At the same time, the presence of 45–60% of cholesterol in such vesicles resulted in nearly instantaneous TTR aggregation [18]. Ali and co-workers showed that not only the chemical structure of the lipids, but also the degree of saturation of fatty acids (FAs) played an important role in TTR aggregation [16,17]. For instance, phosphatidic acid (PA) with unsaturated FAs accelerated TTR aggregation stronger compared to PA with saturated FAs [17]. Similar results were reported by Hou and coworkers [19,20].

Our group showed that lipids affect the aggregation not only TTR, but a large number of amyloidogenic proteins, including α-synuclein (α-syn), insulin, and lysozyme [21–28]. Lipid-assisted misfolding of these protein results in their self-assembly into amyloid oligomers and fibrils [21–26]. Matveyenka and co-authors found that PC strongly inhibited lysozyme and insulin aggregation [21,22,29]. However, anionic lipids, including cardiolipin (CL) and phosphatidylserine (PS), strongly increased the aggregation rate of such proteins [30,31]. The researchers also demonstrated that these lipids if present at the stage of protein aggregation caused drastic changes in the secondary structure of amyloid oligomers and fibrils [21,22,29,32]. These aggregates showed substantially different cytotoxicity compared to the oligomers and fibrils formed in the absence of lipids [33,34]. For instance, insulin oligomers developed in the presence of PCs were less toxic to rat neurons if compared to the oligomers developed in the presence of vesicles composed of PSs and CLs [26,34]. However, amyloid fibrils formed by amyloid β_1–42_, the peptide linked to onset of Alzheimer’s disease, in the presence of LUVs composed of CLs and cholesterol (Cho) showed much greater cell toxicity than the fibrils formed in the lipids-free environment [35]. At the same time, Ivankov and co-workers showed that Aβ triggered membrane disruptions remain unaffected by cholesterol [36]. Jakubec and co-authors found that Cho strongly accelerated fibrillization of α-syn [37], while Dou and co-workers showed that PC decelerated the aggregation rate of this protein [38]. Finally, Frese and co-workers demonstrated that unsaturated PS accelerated the rate of lysozyme aggregation much stronger compared to PS with saturated FAs [39].

These studies suggest that changes in the lipid composition of plasma membranes could be an underlying molecular cause of amyloid diseases. For instance, Matveyenka and co-workers demonstrated that LUVs that are composed of 20% PS, 40% phosphatidylethanolamine (PE), and 40% PC fully inhibited insulin aggregation [40]. At the same time, an increase in the concentration of PS to 30% and 40% causes insulin aggregation and fibril formation [40]. Expanding upon this, we investigate the effect of PC, one of the most abundant phospholipids in the plasma membrane, on TTR aggregation. In most of the cells, PC constitutes ~ 40% of the plasma membrane lipids. Therefore, we prepared LUVs that contain 30% (low), 35% (medium) and 40% (normal) of PC together with PS and PE. It should be noted that the effect LUVs composed of PS and PC alone was previously reported by Ali et al. [16,18] It was found that both DMPS and DMPC decelerated the rate of TTR aggregation [16,18]. However, PE has acceleration effects on TTR aggregation (Fig. S1). In this study, we used thioflavin T (ThT) assay to investigate how different ratios of PC, PE and PS would alter the rate of TTR aggregation. For this, TTR in the presence and absence of LUVs was aggregated at pH 3. We also utilized several biophysical methods, including circular dichroism (CD), Infrared (IR) and nano-Infrared spectroscopy, as well as atomic force microscopy (AFM), to examine the secondary structure and morphology of TTR aggregates. Finally, we employed lactate dehydrogenase (LDH) assay to determine the extent to which different concentrations of PC in LUVs would alter the toxicity of TTR fibrils.

## 2. Results and discussion

### 2.1. Rate of TTR aggregation depends on the concentration of PC in the membranes

**Fig. 1.**
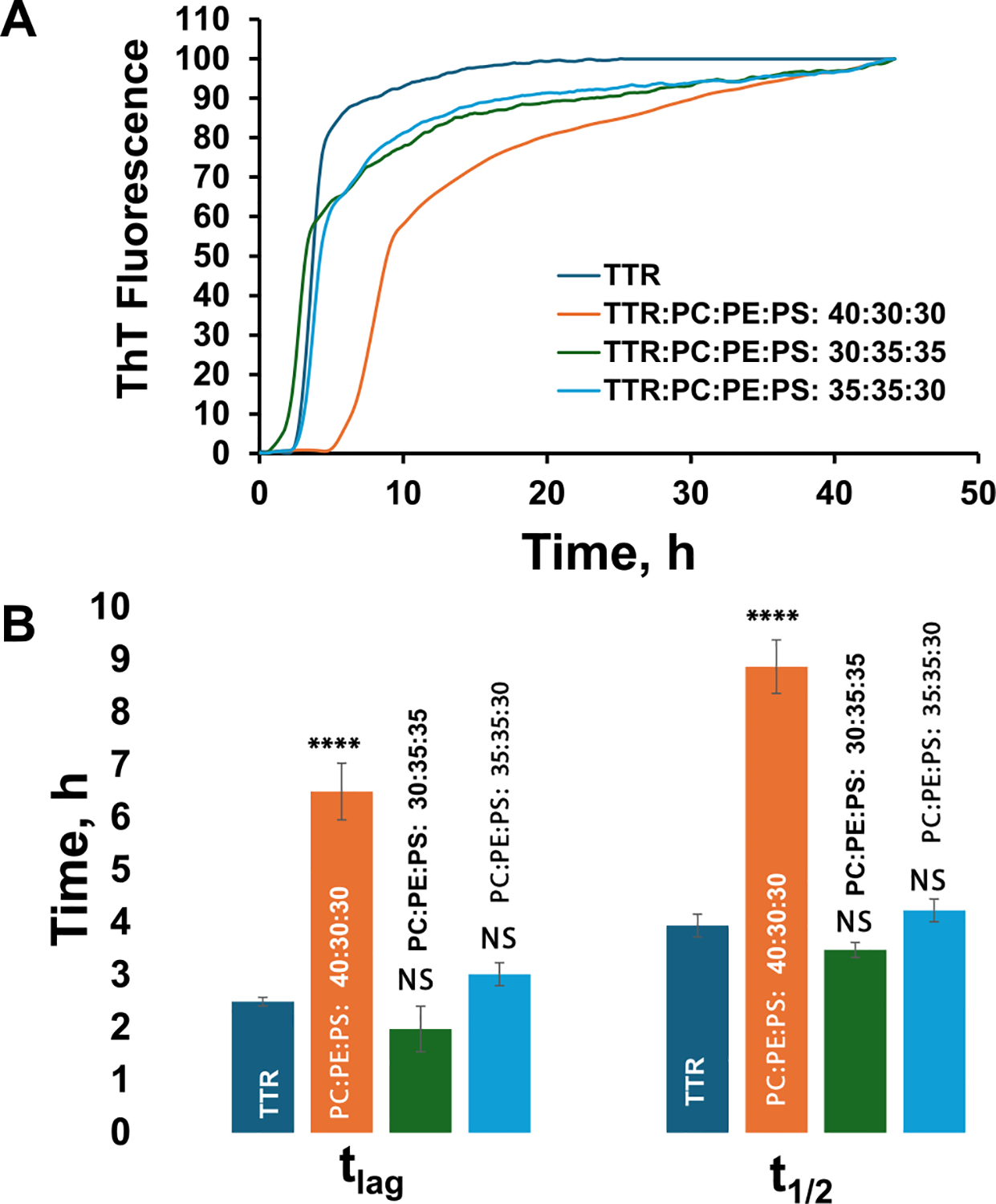
ThT aggregation kinetics of TTR in the lipid-free environment, as well as in the presence of LUVs containing 30%, 35% and 40% PC (A). Each kinetic curve is the average of three independent measurements. Corresponding bar graphs (B) show t_lag_ and t_1/2_, which correspond to 10% and 50% of ThT intensity, respectively. The graphical data are presented as the mean ± SEM. According to one-way ANOVA followed by Tukey’s HSD test, *p < 0.05, **p < 0.01, ***p < 0.001, *p* < 0.0001. NS- non-significant difference.

ThT assay revealed that under the lipid-free conditions, TTR aggregated with a lag-phase (t_lag_) of 2.49 ± 0.08 h, Fig. 1. The lag-phase is followed by a fast rise of ThT fluorescence. This fast increase in the dye fluorescence indicated the appearance of amyloid fibrils. We found that the presence of LUVs with 40% PC, which corresponds to the normal level of PC in the plasma membranes, drastically decelerated TTR aggregation increasing both t_lag_ (6.48 ± 0.53 h) and a half-time (t_1/2_ = 8.86 ± 0.51 h), Fig. 1. At the same time, a decrease in the concentration of PC to 35% and 30% results in TTR aggregation with t_lag_ of 1.97 ± 0.43 h and 1.85 ± 0.081 h, respectively. These results indicate that a decrease in the concentration of PC in the lipid membranes strongly accelerated TTR aggregation. We also found that changes in the concentration of both PE and PS in the LUVs from 30% to 35% did not cause substantial changes in the aggregation rate of TTR. These results indicate that PC uniquely stabilizes TTR, while this effect is not evident for PE and PS. Finally, if the concentration of PC in LUVs was increased to 45% and 50%, no changes in the aggregation rate compared to 40% PC were observed (Fig. S2). It should be noted that LUVs composed of 100% PC caused the same (7.23 ± 1 h) delay of TTR aggregation as LUVs composed of 40% PC [18]. Thus, we can conclude that the concentration effect of PC on TTR aggregation has a plateau that is reached at 40%.

### 2.2. Morphological analysis of protein aggregates

**Fig. 2.**
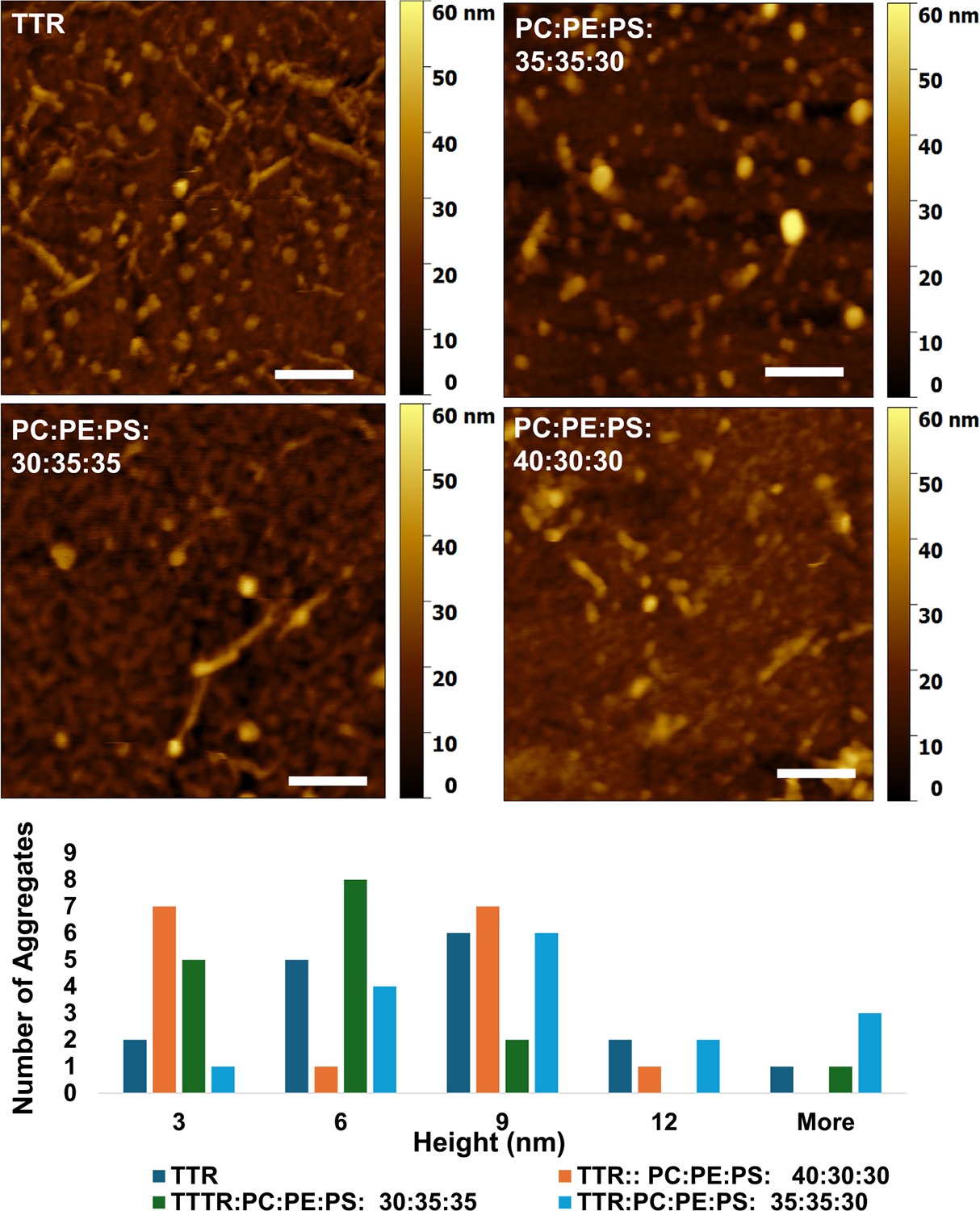
AFM images (top) of TTR aggregates grown in the lipid-free environment and in the presence of LUVs with different concentrations of PC. All images correspond to 48 h of protein aggregation at 37 °C. The scale bar is 500 nm. Histograms (bottom) of height distribution of the observed aggregates. On average, 25 individual protein aggregates were measured in each sample.

We found that in the absence of LUVs, TTR forms thin fibrils with an average height of 6 nm together with small spherical oligomers, Fig. 2. Morphologically similar aggregates were observed in the sample that contained TTR and LUVs with 40% PC. These fibrils were 3–12 nm in height, Fig. 2. We found that a decrease in the concentration of PC in LUVs to 30% caused the formation of thin worm-like fibrils together with thick fibrillar species. Finally, the presence of LUVs with 35% of PC resulted in the formation of short fibrillar species that had 6–9 nm in height. These results indicate that concentration of PC in the lipid membranes uniquely alters the morphology of TTR aggregates.

### 2.3. Structural analysis of TTR fibrils formed in the presence of LUVs with different concentrations of PC

To examine the secondary structure of TTR aggregates, we employed atomic force microscopy Infrared (AFM-IR) spectroscopy [41–43]. This technique allows for landing the scanning probe directly at the surface of the protein aggregate [44–46]. Next, pulsed IR light illuminates the samples’ surface causing thermal expansions in the aggregates present on it. The expansions are recorded by the tip [47–50]. Finally, the thermal expansions are converted into IR spectrum which can be used to examine the secondary structure of the aggregates. In this case, fitting of amide I band can be used. A shift of the amide I band to ~ 1625 cm^−1^ indicates parallel-β sheet, whereas its red-shift to 1659 cm^−1^ indicates the predominance of unordered secondary structure in the analyzed samples. A further redshift of the amide I to ~1697 cm^−1^ indicates the presence of anti-parallel β-sheet.

**Fig. 3.**
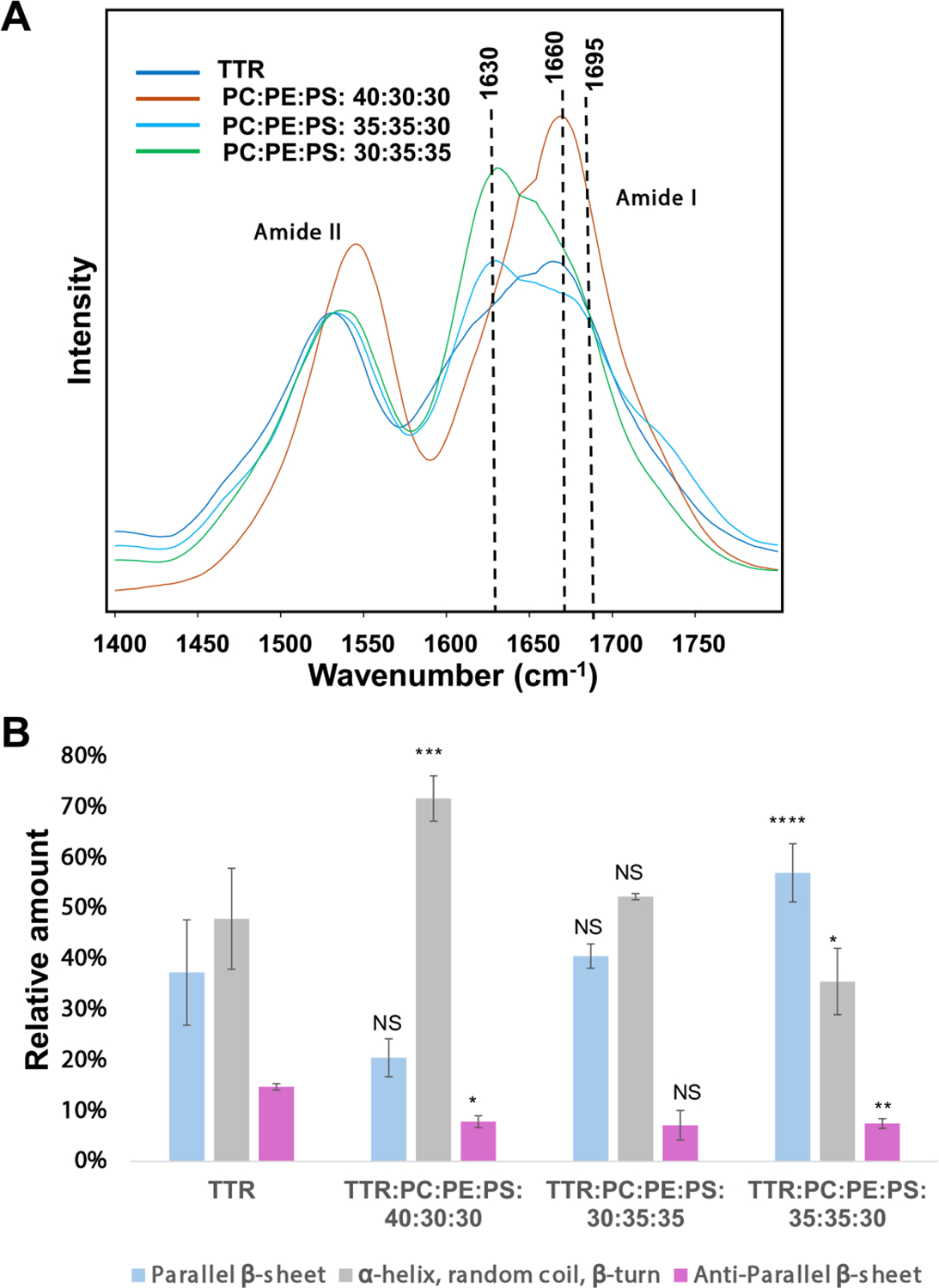
Averaged AFM-IR spectra acquired from TTR fibrils formed in the presence of LUVs with different amount of PC, as well as in the lipid-free conditions (TTR). Corresponding bar graphs that summarize the secondary structure in aggregates based on the fitting results of the amide I band. β-Sheet (parallel, 1632 cm^−1^) in light blue, α-helix and random coil (1660 cm^−1^) in grey, β-sheet (anti-parallel, 1674 cm^−1^) in pink. The graphical data are presented as the mean ± SEM. According to one-way ANOVA followed by Tukey’s HSD test, *p < 0.05, **p < 0.01, ***p < 0.001, *p* < 0.0001. NS- non-significant difference.

We found that TTR fibrils grown in the lipid-free environment, had ~ 30% of parallel β-sheet and ~ 40% of unordered protein, Fig. 3. TTR fibrils that were developed with 40% PC, were dominated by unordered protein, which indicates that the presence of PC prevents the aggregation of TTR into parallel β-sheet. Low concentrations of PC in the lipid membranes (30%), did not result in significant changes in the secondary structure of TTR fibrils. However, we found that TTR fibrils possessed significantly higher and lower amounts of parallel and anti-parallel β-sheet, respectively. These results indicate that small changes in the concentration of PC results in substantial differences in the secondary structure of fibrils formed by TTR in the presence of such lipid membranes.

It should be noted that such important structural information cannot be obtained using traditional FTIR and CD, Fig. S3 and S4. Both FTIR and CD probe the bulk volume of analyzed samples. Since such samples may have unaggregated protein, the acquired FTIR and CD signals would come from a variety of protein aggregates present in each sample and unaggregated protein. Therefore, these techniques cannot be used to examine the secondary structure of individual protein aggregates. Using CD, we found that all acquired spectra had minima at ~ 222 nm and maxima at ~ 203 nm, which corresponds to β-sheet, Fig. S3. FTIR spectra acquired from all analyzed samples exhibited amide I band centered at 1630 cm^−1^ which indicates the dominance of the parallel β-sheet in the protein aggregates observed in these samples, Fig. S4.

### 2.4. Toxicity of protein aggregates formed in the presence of LUVs with 30–40% concentrations of PC

**Fig. 4.**
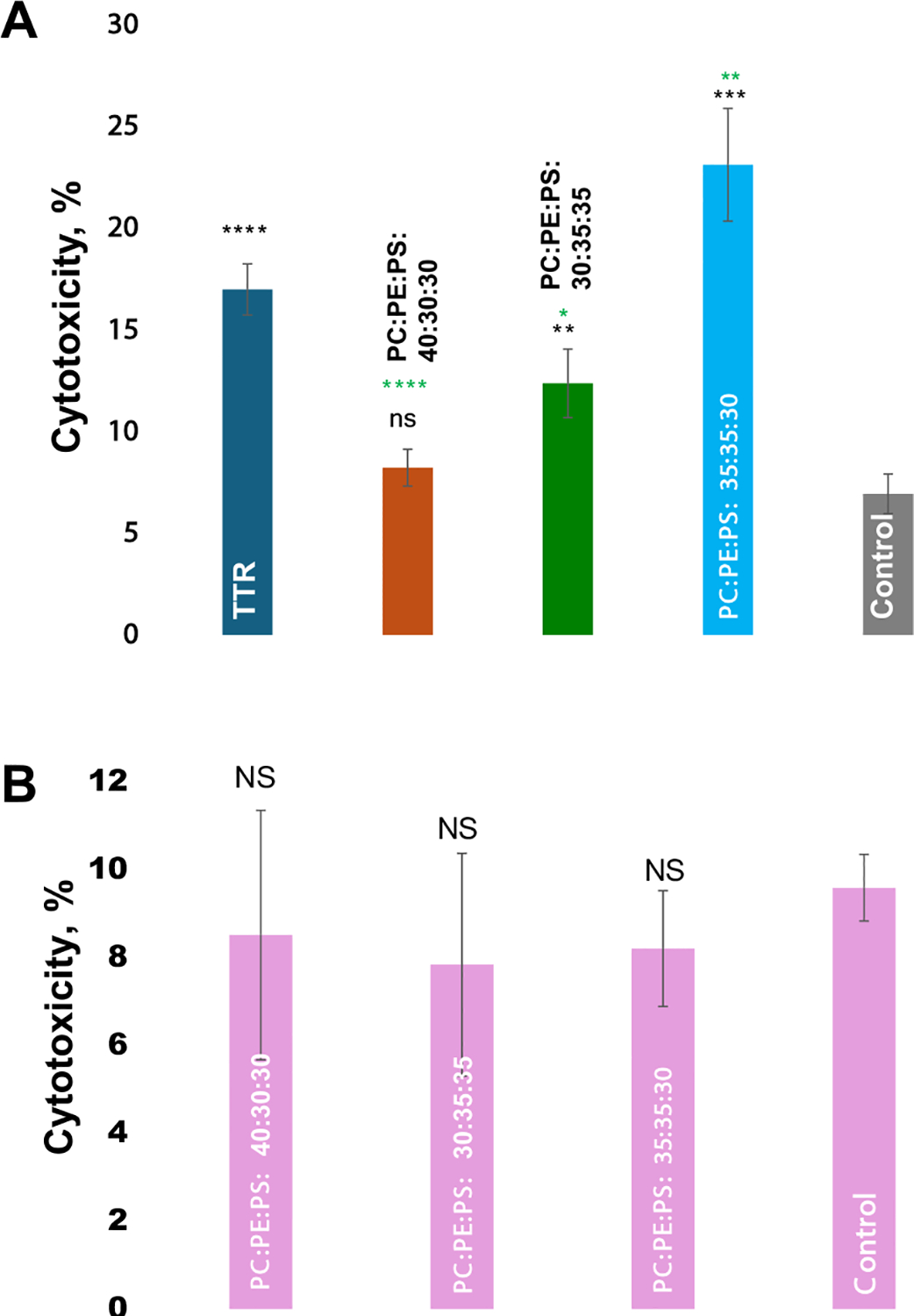
Toxicity of TTR aggregates grown in the presence of LUVs with different amount of PC. Histograms of LDH toxicity assays of (A) TTR fibrils formed in the absence of lipids (TTR), in the presence of LUVs with 40%, 35% and 30% PC as well as (B) LUVs themselves. The graphical data are presented as the mean ± SEM. According to one-way ANOVA followed by Tukey’s HSD test, *p < 0.05, **p < 0.01, ***p < 0.001, *p* < 0.0001. NS- non-significant difference.

We utilized LDH assay to determine the extent to which the concentration of PC in LUVs could alter the toxicity of TTR fibrils. We found that TTR fibrils grown in the presence of LUVs with 40% PC, were not toxic (8.2%) to neuronal cells, Fig. 4. The presence of LUVs with 30% PC, lowered the toxicity of TTR fibrils (12.4%), whereas protein aggregates that were formed in the presence of LUVs with 35% of PC were more toxic (23.1%) compared to TTR fibrils formed in the lipid-free environment (17.0%). Previously, we demonstrated that toxicity of amyloid fibrils directly correlates with the concentration of β-sheet (parallel) in their secondary structure [21,35]. Finally, we found that LUVs themselves were not toxic to neurons, Fig. 4. These results strongly support this phenomenon. Specifically, we found that with a decrease in the amount of parallel β-sheet, the toxicity of TTR aggregates decreased, while an increase in the ratio of parallel β-sheet to other structures resulted in the increase in the toxicity of TTR fibrils. Based on these results, we can conclude that concentration of PC altered the amount of parallel β-sheet, which in turn, changed the toxicity of TTR fibrils.

Critical assessment of experimental results suggests that an increase in the concentration of PC in lipid vesicles results in a linear increase in both t_lag_ and t_1/2_, Table 1. We did not observe a linear increase in the amount of unordered secondary and cytotoxicity that TTR aggregates exerted to rat dopaminergic neurons, Table 1, as the concentration of PC in LUVs changed. At the same time, we observed a direct relationship between the amount of unordered protein and cytotoxicity of TTR aggregates. These results indicate that an increase in the concentration of PC in LUVs only alters the aggregation rate of TTR and does not result in the changes in the secondary structure of TTR aggregates formed in the presence of such LUVs. These findings are consistent with the previously reported results by our group [21,22,40,51] Specifically, Zhaliazka and co-workers found that changes in protein-lipid ratio only changed kinetics of lysozyme aggregation causing no effect on the secondary structure and toxicity of lysozyme fibrils [51], while Matveyenka and co-workers demonstrated that an increase in PC in LUVs only altered the aggregation rate of insulin without substantial changes in the secondary structure and toxicity of insulin fibrils [40].

Finally, it is important to note that experimental results reported in our study demonstrate that transthyretin amyloidosis, amyloid cardiomyopathy and polyneuropathy could be linked to changes in the lipid profile of neuronal or cardiomyocyte cells, which in turn, facilitate TTR aggregation [52]. Nevertheless, experimental validation of these findings at physiological pH is required to establish a clear connection between changes in the lipid composition of plasma membranes and TTR pathologies. Furthermore, it is important to note that the current work, which was focused only on three different lipid mixtures, is insufficient to fully understand pathological processes linked to TTR aggregation. Future studies are required to test changes in the concentration of PS, PE and other lipids in the context of pathological TTR aggregation. In our previous studies, we showed that not only concentration of a particular lipid, but also the length and saturation of its fatty acid play an important role in protein aggregation [15,16,18,53]. Therefore, it becomes important to understand the relationship between changes in the concentration of such lipids at TTR aggregation, which is the subject of separate studies.

## 3. Conclusions

Summarizing, our findings demonstrate that the concentration of PC in lipid membranes plays an important role in stabilization of TTR. We found that TTR stability in the presence of LUVs with physiological concentration of PC increased compared to the low concentrations of this important phospholipid. These results suggest that changes in the lipid concentration of plasma membranes can be an important factor that triggers TTR aggregation. Our results also showed that a decrease in the concentration of PC increased the rate of protein aggregation. At the same time, changes in the concentration of PC in LUVs do not correlate with changes in the secondary structure and toxicity of TTR fibrils. At the same time, we observed a direct relationship between the amount of unordered protein secondary structure and toxicity of fibrils formed in the presence of LUVs. Specifically, the greater the amount of unordered protein, the lower is cytotoxicity of protein aggregates. These findings are consistent with previously reported results for insulin and lysozyme aggregates formed in the presence of LUVs [21,22]. Finally, these findings suggest that PC can be used as a therapeutic against the progression of transthyretin amyloidosis.

## 4. Materials and methods

Lipids: 1,2-dimyristoyl-glycero-3-phospho-l-serine (DMPC), PC, 1,2-dimyristoyl-sn-glycero-3-phospho-l-serine (DMPS), PS and 1,2-dimyristoyl-sn-glycero-3-phosphoethanolamine (DMPE), PE were purchased from Avanti Polar Lipids. ThT, was purchased from Sigma (St. Louis, MO, USA), GVS Cellulose Acetate (CA, USA) Filtration Membrane was purchased GVS technology (Italy).

LUVs preparation: PC, PE and PS were mixed according to the composition of LUVs and dissolved in chloroform. After the solvent was evaporated, lipid film was re-solubilized in PBS. Next, solutions were subjected to six cycles of flash freezing and thawing, followed by sonication. The resulting lipid suspension was then extruded through a 100 nm polycarbonate membrane three times to ensure uniform vesicle size. The size distribution of the lipid vesicles was assessed using dynamic light scattering (DLS) on a DynoPro NanoStar II (Wyatt Technology, Waters Corporation). It should be noted that such LUVs remain stable under acidic pH over several hours, Figure S5 [40].

Cloning of TTR: A plasmid encoding the TTR amino acid sequence (pcDNA3.1+/C-(K) DYK; Accession No. NM_000371) was obtained from GenScript USA. The TTR gene was cloned following the procedure described by Ali et al., The pcDNA3.1+/C-(K) plasmid was amplified by PCR using primers designed with restriction sites: the forward primer 5′-ATATATAAGCTTATGGACTACAAAGACGATGACGACAA-GATGGCTTCTCATCGTCTG-3′ was tagged with a *Hin*dIII site, and the reverse primer 5′-ATATATCTCGAGTCATTCCTTGGGATTGG-3′ was tagged with an *Xho*I site. This resulted in the amplification of a construct encoding the mature TTR protein without its signal sequence. The amplified PCR product and the pET28b vector (GenScript) were digested with *Hin*dIII and XhoI. After digestion, the insert (TTR gene) and the vector were ligated using T4 DNA ligase. The ligation product was transformed into E. coliDH5α cells, which were plated on LB agar plates containing 50 μg/mL kanamycin (Km). Positive transformants were selected and plasmid DNA was extracted using GeneJet Minipreps (Thermo Scientific, USA). Correct cloning was confirmed by restriction digestion with HindIII and XhoI (New England Biolabs), followed by sequencing (Eurofins).

Protein Expression and Purification: TTR expression was induced in *Escherichia coli* BL21 (DE3) cells following the method by Volles and Lansbury [54]. Bacterial cultures in LB broth were induced with 1 mM IPTG, and cells were harvested by centrifugation. The cell pellet was resuspended in a lysis buffer (50 mM Tris, 150 mM NaCl, 10 mM EDTA, pH 8.0) containing a protease inhibitor cocktail (Roche). The suspension was sonicated and subsequently heated in a boiling water bath for 20 min. After centrifugation (16,000 *g*, 30 min), the supernatant was exposed to streptomycin sulfate (136 μL/mL) and glacial acetic acid (228 μL/mL). The mixture was centrifuged again (16,000 *g*, 4 °C, 10 min). The precipitated protein was exposed to a solution of saturated ammonium sulfate and water premixed at 1:1, *v*/v ratio. The protein pellet was resuspended in a solution that contained 100 mM ammonium acetate. Next, the mixture was stirred for 10 min, and further precipitated using absolute ethanol. The final pellet was resuspended in 100 mM ammonium acetate, lyophilized, and stored at − 20 °C for future use.

Size Exclusion Chromatography (SEC): The purified TTR was kept in PBS buffer (pH 7.4) and incubated with thrombin protease overnight at 4 °C to cleave the His-tag. The cleavage reaction was centrifuged (14,000 *g*, 30 min) to remove any aggregates. The clarified TTR solution was then concentrated, and 500 μL of the solution was loaded onto a Superdex 200 Increase 10/300 gel filtration column attached to an AKTA Pure system (GE Healthcare). The column was equilibrated with PBS, and the protein was eluted at a flow rate of 0.5 mL/min at 4 °C.

Protein Aggregation: In a lipid-free environment, TTR (50 μM) was dissolved in 0.5 M sodium acetate buffer containing 1 M KCl, and the pH was adjusted to 3.0 using concentrated HCl and NaOH [10,11]. For aggregation studies with LUVs, 50 μM TTR was mixed with an equimolar concentration of the corresponding lipids (50 μM), and the pH was similarly adjusted to 3.0. Samples were then incubated in a heat block at 37 °C with continuous agitation at 510 rpm for 48 h.

Thioflavin T (ThT) assay: Kinetic measurements of protein aggregation were performed using Spark Tecan plate reader. After samples were added to a well-plate and mixed with ThT, samples were agitated at 510 rpm under 37 °C for 48 h. Measurements were made every 20 min with 450 nm excitation at 488 nm emission. In all experiments, 1 mM stock solution of ThT in H_2_O was used. Prior to experiments, solution was passed through 0.2 μm syringe filter and diluted with PBS (pH 7.4) to the final concentration in each well of 25 μM.

Atomic Force Microscopy (AFM) Imaging: To analyze the morphology of TTR aggregates formed after 48 h of aggregation, AFM was performed using an AIST-NT-HORIBA system (Edison, NJ, USA) with tapping-mode AFM probes that had a force constant of 2.7 N/m and a resonance frequency of 50–80 kHz were used. For each sample, an aliquot was diluted in deionized water in 1:2 *v*/v ratios and applied to a pre-cleaned glass coverslip. After a 20–30 min incubation, excess solution was removed, and the coverslips were dried under nitrogen flow. AFM images were processed using AIST-NT software.

Circular Dichroism (CD): After 48 h of incubation at 37 °C, the protein samples were diluted by DI water and analyzed using a Jasco J-1000 CD spectrometer (Jasco, Easton, USA). Spectra were recorded within 190–250 nm, with three scans per sample, and averaged. Prior to recording the CD spectra, the samples were diluted in PBS (pH 7.4) at a 1:5 ratio. After dilution, the samples contained 10 μm aggregates consisting of both protein and lipid components.

Attenuated Total Reflectance Fourier-Transform Infrared (ATR-FTIR) Spectroscopy: After 48 h of incubation, the protein samples were dried at room temperature on an ATR crystal, and spectra were collected using a Perkin-Elmer 100 FTIR spectrometer (Waltham, MA, USA). Each sample was measured three times.

Atomic force microscopy Infrared (AFM-IR) spectroscopy: For the nanoscale structural analysis of TTR fibrils, an aliquot of the sample was deposited on a 70 nm Au pre-coated silicon wafer. After ~20 min of sample exposition, the excess of sample was removed, and the sample was dried. AFM-IR analysis was done using Nano-IR3 system (Bruker-Nano, Santa Barbara, USA). Scanning probes were optimized using a polymethyl methacrylate standard. On average, over 20 structures were measured in each TTR and TTR:lipid sample with cm^−1^/pt. spectral resolution; 1648–1652 cm^−1^ spectral region was removed to account for the chip-to-chip transition artifact of the QCL laser. Using MATLAB, 2 polynomial order Savitzky-Golay smoothing was applied to all spectra. In AFM-IR analysis, we focused on fibrillar species. While fibrillar structures were predominantly observed in all samples, some samples exhibited non-fibrillar or amorphous morphologies. This suggests that the aggregation process under our experimental conditions may promote a mixture of fibrillar and amorphous assemblies. Processed data for each sample were split into 3 averaged spectra, 10 spectra averaged into 1, and applied a baseline correction (level + zero) and conducted peak fitting using GRAMS/AI^™^ Suite ThermoCorp software, Fig. S6. Statistical analysis of peak fitting values for significance analyzed using in-lab MATLAB script, running Anderson-Darling test followed by Kruskal-Wallis test.

Cell Toxicity Assays: Rat midbrain N27 cells were cultured in RPMI 1640 medium (Thermo Fisher Scientific, USA) supplemented with 10% fetal bovine serum (FBS) (Invitrogen, USA). Cells were seeded in 96-well plates at a density of 50,000 cells per well and incubated at 37 °C under 5% CO_2_ until they reached ~ 70% confluency. After 24 h, the medium was replaced with fresh RPMI 1640 containing 5% FBS and protein aggregate samples. Following another 24-h incubation, the cytotoxicity of protein aggregates was assessed using a lactate dehydrogenase (LDH) assay (G1781, Promega, USA). Absorbance was measured at 490 nm using a plate reader (Tecan, Männedorf, Switzerland), with 25 measurements taken per well at different locations; at least 3 wells were tested per sample. Final sample concentration was 5 μm.

## Supplementary Material

1

## Figures and Tables

**Table 1:** Summary of experimental results observed for TTR and TTR aggregated in the presence of LUVs that contained 30%, 35% and 40% PC.

Sample	t_lag_, h	t_1/2_, h	Unordered secondary structure, %	Cytotoxicity, %
TTR	2.49 ± 0.08	3.94 ± 0.42	47.9	17.0
30% PC	1.85 ± 0.08	3.48 ± 0.32	52.3	12.4
35% PC	1.97 ± 0.43	4.23 ± 0.20	35.5	23.1
40% PC	6.48 ± 0.53	8.86 ± 0.51	71.3	8.23

## Data Availability

Data will be made available on request.

## Appendix A. Supplementary data

Supplementary data to this article can be found online at https://doi.org/10.1016/j.bpc.2026.107574.
