# Supplementary material for: Changes in the concentration of phosphatidylcholine in lipid bilayers determines the aggregation rate of transthyretin
Source: Biophys Chem. Author manuscript; Available in PMC 2026 Jun 28. (PMC13310245; doi:10.1016/j.bpc.2026.107574)
Supplement: 1 [file NIHMS2189640-supplement-1.docx]

**Changes in the Concentration of Phosphatidylcholine in Lipid Bilayers Determines the Aggregation Rate of Transthyretin**

Abid Ali^1^, Mikhail Matveyenka^1^ and Dmitry Kurouski^1*^

1. Department of Biochemistry and Biophysics, Texas A&M University, College Station, Texas 77843, United States

Email:dkurouski@tamu.edu

**Supplementary Information**

Figure S1. ThT aggregation kinetics of TTR in the lipid-free environment, as well as in the presence of LUVs containing 100% PE (top). Each kinetic curve is the average of three independent measurements. Corresponding bar graph (bottom) shows t_lag_ and t_1/2_, which correspond to 10% and 50% of ThT intensity, respectively. T-test was used to determine statistical significance in t_lag_ and t_1/2_. *P<0.05; NS is non-significant difference.

Figure S2. ThT aggregation kinetics of TTR in the lipid-free environment, as well as in the presence of LUVs containing 50:25:25 PC:PS:PE and 45:27.5:27.5 PC:PS:PE. Each kinetic curve is the average of three independent measurements. Corresponding bar graph (bottom) shows t_lag_ and t_1/2_, which correspond to 10% and 50% of ThT intensity, respectively. T-test was used to determine statistical significance in t_lag_ and t_1/2_. *P<0.05; NS is non-significant difference.

Figure S3. CD spectra of TTR fibrils formed in the presence of FAs PC:PE:PS: 40:30:30 PC:PE:PS: 35:35:30, PC:PE:PS: 30:35:35 and in the lipid-free environment

Figure S4. FTIR spectra of TTR fibrils formed in the presence of FAs PC:PE:PS: 40:30:30 PC:PE:PS: 35:35:30, PC:PE:PS: 30:35:35 and in the lipid-free environment.

Figure S5. DLS readings of LUVs of lipid mixtures prepared at pH 7.4 (0h) and after 20h at pH 3.0 (20h).

Figure S6. Deconvolution of amide I band in the acquired AFM-IR spectra with the corresponding contributions of different protein secondary structures.
